# Comparison of immediate versus post-stenting ureteroscopy for ureteral stones treatment

**DOI:** 10.25122/jml-2023-0199

**Published:** 2023-12

**Authors:** Ali Hadi Sabhan

**Affiliations:** 1 Department of Surgery, College of Medicine, University of Al-Qadisiyah, Al-Diwaneyah, Iraq

**Keywords:** Double-J, renal colic, stent, ureteral stones, ureteroscopy, PS-URS: Post-Stenting Ureteroscopy, I-URS: Immediate Ureteroscopy, MET: Medical Expulsive Therapy, SWL: Shock Wave Lithotripsy

## Abstract

Ureteroscopy is a highly effective treatment for ureteral stones, characterized by a high stone-free rate and a low need for re-treatment. Ureteral stent placement can improve the insertion of the ureteral access sheath and ureteroscope but may be associated with higher morbidity prior to and after ureteroscopy. The study aimed to compare immediate *versus* post-stenting ureteroscopy for ureteral stone treatment in terms of operative time, intra- and post-operative complications, length of hospital stay, and stone-free rate. This prospective study involved 126 patients with ureteral stones divided into two groups: the post-stenting ureteroscopy group (PS-URS), who underwent primary ureteral stenting by double J followed by delayed ureteroscopy, and the immediate ureteroscopy group (I-URS), who underwent immediate ureteroscopy without previous stenting. Sixty-six patients were included in the PS-URS group and 60 patients in the I-URS group. Results were comparable, with no significant differences between both groups. The mean operative time was 33.77±3.51 minutes for the PS-URS group and 34.60±2.01 minutes for the I-URS group. The average length of hospital stay was 0.84±2.55 days for PS-URS and 0.92±1.96 days for I-URS patients. The stone-free rate was 97% in the PS-URS group and 95% in the I-URS group. The overall complication rate was 4.5% *versus* 5% in the PS-URS and I-URS groups, respectively, with all complications being minor and managed effectively. Immediate ureteroscopy is a safe and relevant operative approach for ureteral stones, with comparative results for post-stenting delayed ureteroscopy.

## INTRODUCTION

Over the past few decades, the incidence of urolithiasis has become more common in both industrialized and developing countries, and this sharp rise is thought to be linked to lifestyle factors such as a decrease in physical activity, dietary patterns, and climate change. About 12% of the population develops urolithiasis. This condition can manifest in individuals of any age, gender, or ethnicity; however, it is more prevalent in men than women, particularly in the age group of 20 to 49 years [1]. The symptoms of a urinary stone depend on its location, which can be in the kidney, ureter, or urinary bladder. Stone generation does not initially create any symptoms. However, as the condition progresses, it may cause attacks of renal colic, hematuria, urine obstruction, urinary tract infections, and hydronephrosis. Stone discomfort can elicit nausea and vomiting as well. Renal colic is the most frequent complaint of patients with ureteral stones presented in the emergency room [2].

Renal stones often remain asymptomatic and undetected, as they frequently do not cause obvious symptoms or functional impairment. On the other hand, ureteral stones are rarely silent and, most of the time, tend to cause discomfort and obstruction. Most renal stones that migrate to the ureter pass on their own. The size of the stone and its position inside the ureter are the two most important parameters influencing the likelihood of stone clearance. Up to 70% of stones with a radius of less than or equal to 6 mm will pass freely [3]. Ureteral stones that do not pass will necessitate surgical intervention. The use of medical expulsive therapy (MET) to facilitate spontaneous stone passage has dramatically altered the presumed course of ureteral stone disease; however, MET is not effective for all ureteral stones. A recent prospective, randomized, single-blind, and multicentric study showed improvement in stone access and stone-free rate with a semi-rigid ureteroscope after using Tamsulosin or Mirabegron for one week before intervention [4].

Initial treatment with shock wave lithotripsy (SWL) or ureteroscopy (URS) is dictated by stone features and position, as well as patient, surgical, and local variables [5]. Ureteroscopy for proximal ureteral stones is accompanied by greater stone-free rates and reduced re-treatment rates than SWL. Initiating treatment with ureteroscopy for larger stones, rather than starting with SWL, is associated with achieving higher and more rapid stone-free rates [6].

URS is generally considered more effective than SWL for treating distal ureteral stones. Various studies have indicated that while SWL and URS achieve similar stone-free rates (SFR), SWL often requires multiple sessions to attain these outcomes. A pneumatic or ultrasonic lithotripter with semirigid ureteroscopy can be suitable for stones in the middle and distal ureter. However, ureteral access above the level of iliac vessels is more challenging due to ureteral tension, which raises the risk of instrument breakage during the procedure. Therefore, flexible ureteroscopes are excellent for proximal ureteral stones to overcome this difficulty [7].

The placement of a ureteral stent before elective URS can aid the insertion of the ureteral access sheath and ureteroscope. Furthermore, it promotes ureteroscopic manipulation of stones, improves stone clearance, and minimizes complications. Stenting may be related to additional morbidity before and after ureteroscopy [8].

An obstructing ureteral stone associated with infection is considered a life-threatening condition. The collecting system should be decompressed immediately since patients who are not drained have a greater mortality rate. In this clinical context, there is no evidence to support the advantage of nephrostomy *versus* stents [9].

Decompression of an obstructed kidney lowers mortality, reduces delays, and can minimize lengthy hospitalizations. The drainage approach should be customized to the patient's clinical situation, stone properties, and capacity of each center. The consensus is that definite therapy should not commence until the occluded system has been decompressed and the infection has been effectively treated [10]. For the treatment of ureteral calculi, most urologists commonly place a ureteral stent after a ureteroscopy. Stent placement post-URS is indicated for several reasons, including ureteral injury, stricture dilation, presence of a solitary renal unit, renal insufficiency, or significant stone burden [8, 11]. Additionally, newer ureteroscopes, characterized by their smaller diameter, greater flexibility, and tapered tips, have reduced the risk of harm during the procedure [11].

The purpose of this study was to compare the results of immediate *versus* post-stenting ureteroscopy for ureteral stone treatment in terms of operative time, intra- and post-operative complications, length of hospital stay, and stone-free rate.

## MATERIAL AND METHODS

This prospective study was conducted between April 2020 and February 2022, including 126 patients (80 men and 46 women)aged between 28 and 65 years, all requiring intervention for ureteral stones. Patients were divided into two groups:

1. **Post stenting ureteroscopy (PS-URS) Group:** This group included 66 patients who underwent initial ureteral stenting with double J stent followed by definitive stone disintegration and removal after two weeks.
2. **Immediate ureteroscopy (I-URS) Group:** This group consisted of 60 patients who received immediate ureteroscopy for stone disintegration and removal.

**Figure 1:**
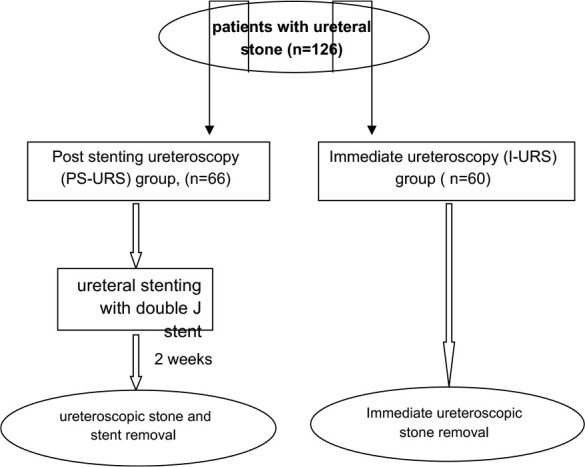
Flow diagram illustrating the strategy of ureteral stone removal in both groups

The specific approaches for ureteral stone removal in each group are detailed in Figure 1. All patients were confirmed to have a single ureteral stone. The evaluation included history taking, physical examination, renal function tests (blood urea, serum creatinine), urinalysis (with or without culture/sensitivity testing), and imaging (abdominopelvic ultrasound, plain X-ray KUB, non-contrast computed tomography scan). Stone characteristics were recorded, including size, site, side, and degree of obstruction caused by the stone (degree of hydronephrosis). Past medical, surgical, and drug histories were checked for all patients before starting any intervention. Inclusion criteria included patients with pain refractory to initial conservative treatment, long-duration obstruction, febrile patients, patients with a single kidney, and the patient’s preference for direct intervention. Febrile and single kidney patients with ureteral stones were included in the PS-URS group, and patients who refused staged intervention were included in the I-URS group. Otherwise, patients were selected randomly between both groups. Sterile urine was achieved, and informed consent was obtained before the intervention. All procedures were performed under general anesthesia with prophylactic antibiotics given at the time of anesthesia induction with either intravenous Ceftriaxone or Amikacin.

In the PS-URS group, a 5-Fr double J stent was inserted using semi-rigid ureteroscopy over a guide wire, with stent placement confirmed by KUB. Two weeks later, a ureteroscopic procedure was carried out for stent removal and stone disintegration. Patients in the I-URS group underwent immediate ureteroscopic stone removal without previous stenting.

Stone disintegration was achieved using pneumatic or laser (holmium: YAG laser, 20 W/100 W) lithotripsy. Stone fragments were extracted using forceps or stone baskets. A semi-rigid 4.5/6.0-Fr ureteroscopy (Karl Storz Endoscopy) was used. Stone-free status was determined intraoperatively, ensuring no fragments were left behind. This status was later confirmed postoperatively through imaging studies. Intraoperative and postoperative complications were managed by standard protocols.

We compared both groups based on several parameters: operative time (recorded during the second procedure in the PS-URS group), intra- and postoperative complications, hospital stay duration, and stone-free rate.

Data analysis was done using SPSS version 18 (Statistical Package for the Social Sciences). Categorical data were evaluated using Chi-square or Fisher's exact tests, while continuous data were assessed using an independent t-test. Statistical significance was defined as a p-value < 0.05.

## RESULTS

This study included 126 patients aged between 28 and 65 years with a solitary ureteral stone. Of these, 66 patients (52%) with a mean age of 40.21±9.54 (46 men and 20 women) were included in the PS-URS group who underwent ureteroscopic stone removal two weeks following primary ureteral stenting, while the I-URS group consisted of 60 patients with a mean age of 42.86±9.84 (34 men and 26 women) who underwent immediate ureteroscopic stone removal. The gender distribution was similar in both groups (p-value=0.14), and there were no significant differences in patients' ages between the two groups (p-value=0.28). Patient characteristics are summarized in Table 1.

**Table 1:** Patient characteristics

Variables		PS-URS group N=66	I-URS group N=60	p-value
Mean age, years		40.21±9.54	42.86±9.84	0.28 ^a^
Gender, n	Male Female	46 20	34 26	0.14 ^b^

a independent-sample t-test; ^b^ Fisher’s exact test

The size of the stones ranged from 8 to 12 mm. The mean stone size was 8.99±1.14 mm in the PS-URS group and 9.44±1.07 mm in the I-URS group, with no significant difference (p-value=0.10). The stone distribution was as follows: in the PS-URS group, right-sided stones were in 30 patients (45.4%) and left-sided in 36 (54.6%). In the I-URS group, right-sided stones were in 20 patients (33.3%) and left-sided in 40 (66.7%). The stone sites were distributed as follows: in the PS-URS group, 20 (30.3%) were upper ureteral, 22 (33.3%) mid-ureteral, and 24 (36.4%) lower ureteral. In the I-URS group, 11 (18.4%) were upper, 23 (38.3%) mid-ureteral, and 26 (43.3%) lower ureteral. Hydronephrosis was mild in 11 patients (16.7%) in the PS-URS group and 9 (15%) in the I-URS group. Moderate hydronephrosis was more common, occurring in 34 patients (51.5%) in the PS-URS group and 40 (66.6%) in the I-URS group. Severe hydronephrosis occurred in 21 (31.8%) and 11 (18.4%) patients in the PS-URS and I-URS groups, respectively. There were no significant differences in stone side (p-value=0.20), site (p-value=0.29), or degree of hydronephrosis (p-value=0.17) between the two groups. The details are displayed in Table 2.

**Table 2:** Stone characteristics

Variables		PS-URS group N=66	I-URS group N=60	p-value
Mean stones size, mm		8.99±1.14	9.44±1.07	0.10 ^a^
Stones side, n (%)	Right	30 (45.4)	20 (33.3)	0.20 ^b^
	Left	36 (54.6)	40 (66.7)	
Stones site, n (%)	Upper	20 (30.3%)	11 (18.4%)	0.29 ^c^
	Mid	22 (33.3)	23 (38.3)	
	Lower	24 (36.4)	26 (43.3)	
Hydronephrosis, n (%)	Mild	11 (16.7)	9 (15)	0.17 ^c^
	Moderate	34 (51.5)	40 (66.6)	
	Severe	21 (31.8)	11 (18.4)	

a independent-sample t-test; ^b^ Fisher’s exact test; ^c^ Chi-square test

The mean operative time in the PS-URS group was 33.77±3.51 minutes compared to 34.60±2.01 minutes in the I-URS group with no statistical difference (p-value=0.55). The mean postoperative hospital stay was 0.84±2.55 days in the PS-URS group and 0.92±1.96 days in the I-URS group, with no significant difference (p-value=0.79). The stone-free rate was comparable between both groups (97% for the PS-URS group and 95% for the I-URS group, p=0.66). Minor complications occurred in three patients in each group (4.5% in PS-URS and 5% in I-URS), including intraoperative bleeding, postoperative pain, and ureteral perforation. The bleeding was resolved spontaneously, the pain was controlled with analgesia, and a double J stent was left after ureteral perforation and removed later without any harm. No patients developed sepsis in the postoperative period. The operative and postoperative statistics are shown in Table 3.

**Table 3:** Operative and postoperative outcomes and complications

Variables	PS-URS group N=66	I-URS group N=60	p-value
Mean operative time, min	33.77±3.51	34.60±2.01	0.55 ^a^
Mean hospital stay, days	0.84±2.55	0.92±1.96	0.79 ^a^
Stone-free rate, %	97	95	0.66 ^b^
Complications, n, (%)	3 (4.5)	3 (5)	0.90 ^c^
Pain	2	2	
Bleeding	1	0	
Perforation	0	1	
Sepsis	0	0	

a independent-sample t-test; ^b^ Fisher’s exact test; ^c^ Chi-square test

## DISCUSSION

In this study, 66 patients were included in the PS-URS group and 60 in the I-URS group. The results were comparable, with no significant statistical differences between groups. The mean operative time was 33.77±3.51 minutes in the PS-URS group compared to 34.60±2.01 minutes in the I-URS group. The average hospital stay was 0.84±2.55 days for the PS-URS group and 0.92±1.96 days for the I-URS group. The stone-free rate was high in both groups, with 97% in the PS-URS and 95% in the I-URS group. The overall complication rate was 4.5% *versus* 5% in the PS-URS and I-URS groups, respectively.

The mean age of patients was 40 years in the PS-URS group and 42 years in the I-URS group. There was a predominantly male gender distribution in this study, with a male-to-female ratio of 2:1. This pattern of age and sex distribution is consistent with other studies [2, 12, 13], reflecting the higher incidence of urolithiasis in men compared to women, particularly between the ages of 20 and 49 [1].

In our study, the mean stone size varied from 7 to 12 mm, averaging 8.9 mm in the PS-URS group and 9.4 mm in the I-URS group. Stones of this size category are less likely to pass spontaneously. Nearly 70% of stones less than or equal to 6 mm in diameter tend to pass spontaneously [3], whereas stones larger than 7 mm show minimal likelihood of spontaneous passage [14]. The most common stone location in both groups was the distal or lower ureter, accounting for 36.4% in the PS-URS group and 43.3% in the I-URS group. This finding aligns with Matani *et al*. [15], who also reported the lower ureter as the predominant site for ureteral stones. Moderate hydronephrosis was the most prevalent degree of obstruction, observed in 51.5% of the PS-URS group and 66.6% of the I-URS group. There was no significant statistical difference between both groups regarding stone size, site, and degree of obstruction (hydronephrosis). The key parameters influencing the likelihood of stone passage are the size and location of the stone inside the ureter [3].

Although the results of post-stenting ureteroscopy are favorable, the statistical analysis showed no significant difference in operative time, postoperative hospital stay, or stone-free rate when comparing immediate *versus* post-primary ureteral stenting ureteroscopy for ureteral stone management. These findings agree with another study comparing emergent *versus* delayed treatment of ureteral stones [16].

The stone-free rate in this study was 95% and 97% in both PS-URS and I-URS groups, respectively. These rates are consistent with the overall ureteroscopic stone clearance rate of 90% for ureteral stones as per the guidelines of the American/European Urological Association [17]. The stone-free rate in our immediate ureteroscopy group (97%) is compared favorably with other studies on emergency ureteroscopy (89–98%) [15, 18, 19].

Furthermore, the mean operative times and postoperative hospital stays in our study groups align with those reported in a larger, related study [20]. The complication rate was 4.5% in the PS-URS group and 5% in the I-URS group, similar to those reported in a global study of complications and outcomes of ureteroscopy [21]. Other studies reported a higher complication rate of 11.8% [22] and 13.1% [19] with emergent ureteroscopy.

Some studies found no added benefit in stone-free or complication rates with routine pre-operative stenting [23]. In contrast, others observed a higher stone-free rate for larger stones with pre-ureteroscopy stenting [24, 25]. Stenting may be accompanied by higher morbidity before and after ureteroscopy, in addition to the detrimental effect of stents on the patient's life and the concerns of encrustation. Therefore, it is recommended that once the decision to remove a stent is made, the removal should ideally occur within two weeks of this decision [8].

In this study, patients with a single kidney who presented with anuria, as well as febrile patients with ureteral stones, were primarily managed with ureteral stenting using double J stents (included in the PS-URS group). A stone-induced obstruction accompanied by infection is a critical condition that demands immediate decompression of the collecting system. Unmanaged cases have a significantly higher mortality rate [9]. Abdel-Kader [26] also reported on the safe practice of emergency ureteroscopy in patients with ureteral stones and anuria.

All the complications of this study were minor and were promptly addressed and treated according to standard protocols. Post-operative pain, the most common complication in both groups, was typically limited to one day and managed effectively with analgesia. In the PS-URS group, one patient experienced minor intraoperative bleeding that resolved spontaneously. In the I-URS group, a simple ureteral perforation occurred in one patient during the removal of a large stone. Following international guidelines [5], a double J stent was placed and removed after four weeks without further complications. None of the patients in our study developed sepsis throughout the postoperative period. Statistical analysis reveals no significant difference in complications between the two groups (p-value=0.90). This low complication rate can be attributed to thorough patient preparation, advancements in endoscopic techniques, and strict adherence to procedural guidelines. Ureteral perforation, a potential intraoperative complication during ureteroscopy, has been reported in the literature with a varied incidence rate of 0% to 14% [27, 28]. The size and location of the stone, operative time, and preoperative infection have been reported as independent predictive factors for ureteral injury [29].

The use of antimicrobial prophylaxis before initiating the intervention has shown a significant reduction in the incidence of postoperative bacteremia [30, 31].

The limitations of this study include a small number of patients. A post-hoc power analysis, assuming a medium effect size and an α-error probability of 0.05, resulted in a power of 0.794. This power increased to 0.99 when considering a large effect size. In addition, the follow-up duration was short, and no cost comparison was made between the two groups.

## CONCLUSION

The immediate ureteroscopic treatment of ureteral stones was both safe and effective. Compared to delayed or post-stenting ureteroscopy, this intervention alleviates patient discomfort, avoids stent-related morbidity, and facilitates quicker recovery without serious complications.
